# PrEParing for Long-acting Technologies: A Multistate Analysis of PrEP Persistence and HIV and STI Lab Coverage Among Oral PrEP Initiators in St. Louis, Missouri (2014–2021)

**DOI:** 10.1093/ofid/ofaf795

**Published:** 2026-01-09

**Authors:** Lindsey M Filiatreau, Rupa Patel, Kate Curoe, Ashley Underwood, Joseph Cherabie, Elvin H Geng, Aditi Ramakrishnan, Rachel Presti, Aaloke Mody

**Affiliations:** School of Public Health, Washington University in St. Louis, St. Louis, Missouri, USA; Community Health Department, Whitman Walker Health, Washington, District of Columbia, USA; Division of Infectious Diseases, School of Medicine, Washington University in St. Louis, St. Louis, Missouri, USA; Division of Infectious Diseases, School of Medicine, Washington University in St. Louis, St. Louis, Missouri, USA; Division of Infectious Diseases, School of Medicine, Washington University in St. Louis, St. Louis, Missouri, USA; Division of Infectious Diseases, School of Medicine, Washington University in St. Louis, St. Louis, Missouri, USA; Division of Infectious Diseases, School of Medicine, Washington University in St. Louis, St. Louis, Missouri, USA; Division of Infectious Diseases, School of Medicine, Washington University in St. Louis, St. Louis, Missouri, USA; Division of Infectious Diseases, School of Medicine, Washington University in St. Louis, St. Louis, Missouri, USA; Division of Infectious Diseases, School of Medicine, Washington University in St. Louis, St. Louis, Missouri, USA

**Keywords:** care cascade, HIV prevention, multistate, PrEP

## Abstract

**Background:**

Engagement in pre-exposure prophylaxis (PrEP) care and routine screening for HIV and sexually transmitted infections (STIs) is critical to realizing the full benefits of this HIV prevention strategy.

**Methods:**

We enrolled a cohort of individuals seeking PrEP services in two clinics in Missouri, an Ending the HIV Epidemic priority state, from June 2014 to November 2021. We used electronic health record and survey data to explore outcomes in the 2 years following linkage to care using multistate methods. We assessed transitions between eight mutually exclusive care states differentiated by receipt of a prescription, lab coverage status, and retention. We describe outcomes in the population overall and distinct sociodemographic subgroups.

**Results:**

A total of 470 individuals were included (90.3% male; median age 29 (IQR, 25–36); 52.9% White, non-Hispanic) and contributed 879.5 person-years of follow-up. One week following linkage to care, 86.8% (95% CI: 83.6–90.0) of participants had a PrEP prescription. At month 6, 35.2% (95% CI: 30.7–39.7) were in care but had a lapse in HIV/STI screening, at month 12, 48.3% (95% CI: 43.7–53.1) were disengaged from care. Of those who disengaged, 28.3% (95% CI: 23.9–32.7) were re-engaged 6 months later. Females and uninsured individuals were the most likely to disengage during the first year of follow-up.

**Conclusions:**

Lapses in clinic visits and lab screening are common among PrEP users in Missouri and most who disengage do not return. Females, uninsured individuals, and other marginalized groups may be particularly susceptible to poor persistence suggesting targeted interventions are warranted.

Key Points In a cohort of PrEP care initiators in Missouri, an EHE priority state, 35% had a lapse in HIV/STI screening 6 months after initiation, ∼50% disengaged from care by month 12, and only 30% of disengagers re-engaged within 6 months.

Pre-exposure prophylaxis (PrEP) is a critical component of the HIV-1 prevention toolbox globally, with some studies suggesting scale-up of PrEP can avert over 30% of incident infections [1]. Yet, individuals with heightened susceptibility to HIV need to first access PrEP and, second, persist on PrEP over time to achieve its maximum benefits [2]. Recently, it was estimated that only 450 000 of the over 2.25 million Americans with a treatment indication were on PrEP [3, 4]; among Americans who initiate PrEP, approximately 25% discontinue by 6 months, 50% by 1 year, and 60% by year 2 following PrEP initiation [5–7]. Evidence also suggests a substantial proportion PrEP users cycle on and off treatment over time (∼20%), demonstrating the challenge many face in persisting on PrEP [8, 9].

In addition to decreasing the risk of HIV, individuals accessing routine PrEP care can experience other benefits from consistent engagement with the healthcare system. For example, PrEP users are routinely screened for HIV and screened and treated (as warranted) for sexually transmitted infections (STIs), including gonorrhea, chlamydia, and syphilis. Yet, poor persistence on PrEP or inconsistent engagement in PrEP care services limit these opportunities for screening and treatment for HIV, gonorrhea, chlamydia, and syphilis. This is particularly critical given that people returning to PrEP care are also known to experience higher rates of STIs [10]. Despite the importance of continued engagement in clinical care and routine screening for STIs and HIV in realizing the full potential of this promising HIV prevention tool, little is known about how individuals initiating PrEP care engage, disengage, and re-engage in services over time and how this influences receipt of guideline-recommended lab testing.

Characterizing individuals' care journeys following PrEP initiation can help identify when and what type of care gaps need to be addressed to optimize sexual health outcomes for PrEP users. Novel longitudinal approaches are gaining traction as a method to better illustrate the dynamic nature of care engagement among people living with a range of chronic conditions, including HIV [11–14]. Among people living with HIV specifically, these methods have been used to highlight unique patterns of care engagement (eg, early vs late disengagement, cyclical vs consistent engagement), which in turn shed light on when and what type of strategies are needed to support sustained care-seeking across the care continuum (eg, at the point of linkage to care, promote reengagement) [15]. Engagement in PrEP care may be similarly variable over time, with individuals cycling in and out of treatment as their behaviors and other circumstances (eg, insurance coverage, competing obligations) may change [16, 17]. Yet, novel longitudinal approaches have not been widely used to understand nuanced patterns of engagement in PrEP care, leaving important gaps in our understanding of the PrEP client journey. Understanding these patterns of engagement will be particularly important as new PrEP technologies (eg, long-acting injectables) are implemented as this can inform which treatment modalities may be most appropriate for distinct users.

In this work, we use multistate methods that can capture the multiple transitions into and out of mutually exclusive and exhaustive clinical care states over time [11, 12, 18] to characterize treatment, care engagement, and appropriate laboratory screening and monitoring over time in a cohort of individuals newly initiating PrEP care in Missouri, an Ending the HIV Epidemic priority state where HIV prevention and care data are limited. Our overarching goal is to capture the dynamic nature of users' care journeys and provide a more nuanced picture of treatment coverage and lab monitoring in the population overall and in distinct subgroups.

## METHODS

### Study Population

Between June 2014 and November 2021, we enrolled individuals initiating PrEP care at two infectious disease clinics in St. Louis City in a longitudinal PrEP cohort. To be eligible, individuals had to be 18 years or older and prescribed emtricitabine/tenofovir disoproxil fumarate (Truvada), emtricitabine/tenofovir alafenamide (Descovy), or Cabotegravir (Apretude) for PrEP from one of the two participating clinics. Participants were recruited by the study team either in person, during a clinic visit, or over the phone. Flyers were posted in each clinic to promote recruitment. When possible, consent was conducted in person. However, due to service delivery interruptions during the COVID-19 pandemic, consent was also sought telephonically when in-person visits were infeasible. Individuals transferring care from other clinics and those screening HIV positive at care initiation were excluded.

### Data Sources

We harnessed electronic health records (EHR) and participant surveys collected at each PrEP care visit using RedCap, a secure web application for collecting survey data, to explore outcomes over time. Specifically, we obtained demographic, care initiation, and treatment initiation data from visit surveys. We obtained PrEP clinic visit dates and laboratory testing data—including all HIV, syphilis, chlamydia, and gonorrhea test dates from the EHR database.

### Measures

**Figure 1.**
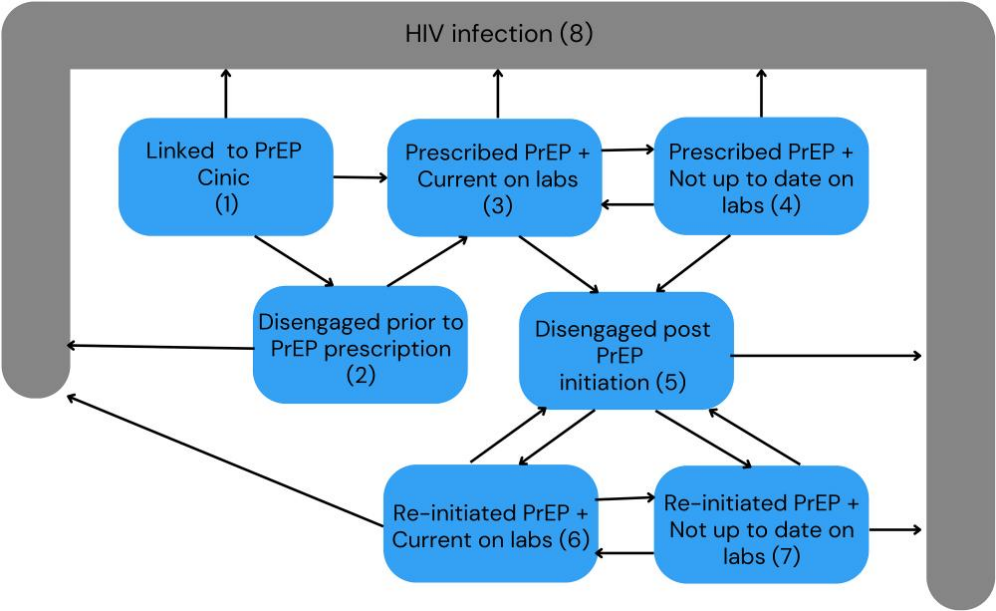
Multistate diagram depicting mutually exclusive and exhaustive PrEP care continuum states and possible transitions between states.

We identified eight mutually exclusive and exhaustive PrEP care-seeking states (Figure 1)—seven nonabsorbing states (ie, states individuals could transition into or out of) and one absorbing state (ie, states that individuals could transition into but not out of). These states were created based on linkage to the clinic, receipt of a PrEP prescription, lab status, clinic engagement, and seroconversion.

*Linkage to PrEP clinic* (nonabsorbing): Individuals were considered linked to the PrEP clinic on the date of their intake visit, as documented in the PrEP cohort survey data.

*Prescribed PrEP* (nonabsorbing): Individuals were considered to be prescribed PrEP the first date a PrEP prescription was documented in their cohort survey data. Data on prescription fills were unavailable.

*Lab status* (nonabsorbing): All individuals entered the cohort as “up to date on labs.” Subsequent to cohort entry, individuals were considered “not up to date on labs” when they experienced a gap of greater than 183 days in gonorrhea/chlamydia, syphilis, or HIV testing, as documented in EHR data. Subsequently, individuals were considered “up to date on labs” on the first date all labs were current.

*Clinic engagement* (nonabsorbing): Individuals who had a gap of greater than 183 days in EHR-documented clinic visits (ie, a 6-month gap in clinic visits) were considered to have “disengaged” from the clinic on the first date the definition was met (ie, on day 184 following their most recent clinic visit). This definition is consistent with that of other studies exploring disengagement from PrEP care [19, 20]. Subsequent to any instance of disengagement from care, individuals were considered “re-engaged at the clinic and reinitiated on PrEP” the first date they returned to the PrEP clinic.

*Seroconversion* (nonabsorbing): Individuals were considered to have seroconverted on the first date of a confirmed HIV positive test result.

We also created six composite outcomes. *Disengaged from the PrEP clinic* included all individuals who experienced a gap in clinic visits of more than 183 days, irrespective of receipt of a PrEP prescription (ie, state 2 + state 5; Figure 1). *Prescribed PrEP and continuously in care* included all individuals who had been prescribed PrEP and had no prior interruption in clinic visits, irrespective of lab status (ie, state 3 + state 4; Figure 1). *Re-engaged in care at the PrEP clinic* included all individuals who had returned to the clinic after a previous lapse in care, irrespective of lab status (ie, state 6 + state 7; Figure 1). *In care and current on labs* included all individuals who were engaged or re-engaged in care and had no gap in routine lab coverage (ie, state 3 + state 6; Figure 1). *In care and late on labs* included all individuals who were engaged or re-engaged in care but had a gap in routine lab coverage of greater than 183 days (ie, state 4 + state 7; Figure 1). Finally, *engaged or re-engaged in care at the PrEP clinic* included all individuals who were not actively disengaged from the clinic, irrespective of lab status (ie, state 3 + state 4 + state 6 + state 7).

*Demographics:* Data regarding participants' sex assigned at birth (male/female), age, race/ethnicity (White, non-Hispanic/Black, non-Hispanic/Hispanic/Asian/Other race/ethnicity), relationship status (Married or partnered/Single, never married/Other), level of education (≤High School/College/Graduate+/Other), employment status (Unemployed/Employed/Student/Other), and insurance coverage (any/none) were determined via self-report. Response options were reported as indicated above for descriptive analyses but were further condensed for sub-group analyses to account for small cell sizes.

### Statistical Analyses

Descriptive statistics (counts and proportions) were used to characterize the study population overall. To quantify longitudinal care continuum outcomes, we merged PrEP cohort data with EHR data using medical record numbers. We then categorized PrEP users in one of eight mutually exclusive and exhaustive care states (Figure 1) at each time point in the 2 years following linkage to care. Overall, there were 20 possible transitions between each of the eight identified care states (Figure 1).

We examined longitudinal care engagement using multiple methods. First, we used nonparametric multistate analytic techniques that employ the Aalen-Johansen estimator to account for competing events and multiple transitions between clinical care states over time [18, 21, 22]. We estimated the probability of a PrEP user being in a particular care state following linkage to PrEP care and from the first instance of disengagement from care following receipt of a PrEP prescription. For these analyses, date of cohort entry and date of first disengagement from PrEP care following receipt of a prescription served as time zero, respectively. Participants were censored at the end of the observation period (1 December 2021). We also estimated the restricted mean time spent in each care state in the 2 years following linkage to PrEP care (ie, estimated time spent in each state out of the 2 years period) [23] and the prevalence of disengagement from care following PrEP initiation stratified by demographic subgroups. For all estimates, 95% confidence intervals were obtained through bootstrapping with 1000 nonparametric resamples of the data.

Data cleaning was conducted in SAS 9.4 (SAS Institute Inc., Cary, NC) and all analyses were conducted in STATA 17 (StataCorp LLC, College Station, TX) and R (R Foundation for Statistical Computing, Vienna, Austria).

### Ethics

This study was approved by Washington University in St. Louis' Institutional Review Board.

## RESULTS

### Participant Characteristics

Of the 516 individuals enrolled in the cohort between June 2014 and November 2021, 470 (90.9%) were included in the analytic dataset and contributed 879.5 person-years of follow-up. Forty-three individuals were excluded because they initiated PrEP at another clinic and two were excluded because they screened positive for HIV at their first visit. Of those included, a majority were assigned male at birth (*N* = 427; 91.4%), White, non-Hispanic (*N* = 243; 52.0%), and single (346; 74.4%) (Supplementary Table 1). A total of 391 individuals (86.9%) were insured, and most were employed (*N* = 295; 67.7%) and had completed at least some college (*N* = 306; 70.3%) (Supplementary Table 1).

### Multistate Analysis Results

**Figure 2.**
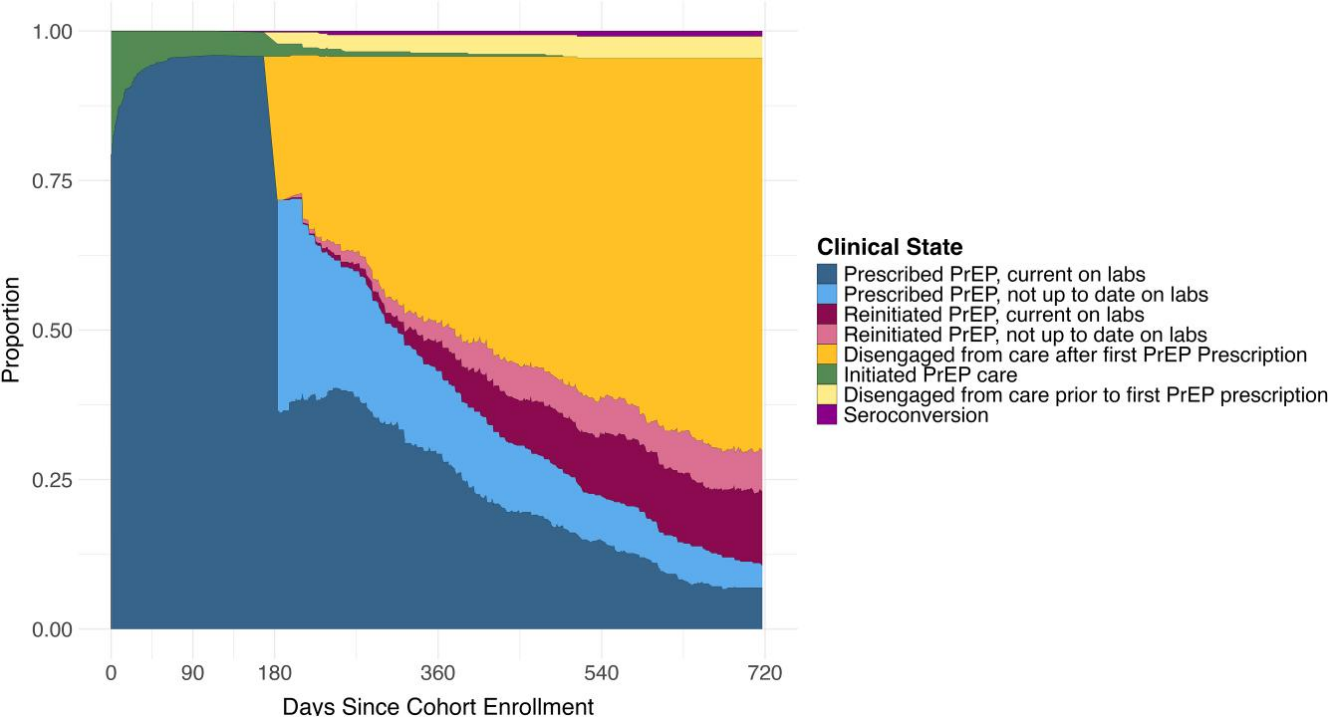
Clinical care outcomes across the first 2 years following linkage to PrEP care in a cohort of 470 individuals linked to PrEP care in St. Louis Missouri between June 2014 and November 2021.

A majority of PrEP users (79.4%; 95% CI: 75.5–83.2) were prescribed PrEP the same day they were enrolled into care, with 93.0% (95% CI: 90.4–95.1) receiving a prescription within 30 days (Figure 2; Table 1). At month six following linkage to PrEP care, 25.9% (95% CI: 21.8–29.8) of the cohort had disengaged from the PrEP clinic (Table 2) and over a third (35.2% [95% CI: 30.7–39.7]) were in care but had a lapse in lab coverage (Table 1). One year following linkage, 48.3% (95% CI: 43.7–53.1) of individuals were disengaged from the PrEP clinic (3.0% disengaged prior to receipt of a prescription [95% CI: 1.5–4.7] and 45.3% disengaged following receipt of a prescription [95% CI: 40.7–50.2]) and 16.8% (95% CI: 13.4–20.4) were in care but had a lapse in lab coverage (Tables 1 and 2). Two years following linkage, 29.0% of individuals were engaged in care (10.6% [95% CI: 7.6–13.4] continuously engaged and 18.5% [95% CI: 15.0–22.5] re-engaged), 70.0% (95% CI: 65.7–74.6) were disengaged, and ∼1% had seroconverted (95% CI: 0.2–1.8; Tables 1 and 2). A total of 11.0% (95% CI: 8.4–14.2) of the population was in care but had a lapse in lab coverage (Table 2). There were 234 positive STI tests—33 syphilis tests, 94 gonorrhea tests, and 107 chlamydia tests—during the first 2 years following linkage to PrEP care. Diagnoses were incurred among 119 distinct PrEP users. Sixty-seven STIs (28.6% of diagnoses) incurred among 49 individuals (41.2% of PrEP users with any STI diagnosis) were diagnosed at the time of linkage to PrEP care; 24 STI diagnoses (10.3% of diagnoses) incurred among 19 individuals (16.0% of PrEP users with any STI diagnosis) occurred upon return to clinical care, immediately following a clinical lapse; 143 diagnoses (61.1% of diagnoses) incurred among 78 individuals (65.5% of PrEP users with any STI diagnosis) occurred while individuals were actively engaged in care during routine STI screening (proportion of PrEP users does not sum to 100.0% as some individuals had diagnoses at entry, upon return, and during continuous engagement; data not shown).

**Table 1.** Proportion of Individuals in Each Mutually Exclusive and Exhaustive PrEP Care Continuum State in the First 2 Years Following Linkage to PrEP Care

Time (d)	Linked to PrEP Clinic (1)	Disengaged From Care Prior to PrEP Prescription (2)	Prescribed PrEP and Current on Routine Labs (3)	Prescribed PrEP and Late for Routine Labs (4)	Disengaged From PrEP Clinic Following PrEP Initiation (5)	Reinitiated on PrEP and Current On Routine Labs (6)	Reinitiated On PrEP and Late on Routine Labs (7)	Seroconverted (8)
0	20.6 (17.0–24.7)	0.0 (0.0–0.0)	79.4 (75.3–83.0)	0.0 (0.0–0.0)	0.0 (0.0–0.0)	0.0 (0.0–0.0)	0.0 (0.0–0.0)	0.0 (0.0–0.0)
7	13.2 (10.4–16.4)	0.0 (0.0–0.0)	86.8 (83.6–89.6)	0.0 (0.0–0.0)	0.0 (0.0–0.0)	0.0 (0.0–0.0)	0.0 (0.0–0.0)	0.0 (0.0–0.0)
30	7.0 (4.9–9.4)	0.0 (0.0–0.0)	93.0 (90.6–95.1)	0.0 (0.0–0.0)	0.0 (0.0–0.0)	0.0 (0.0–0.0)	0.0 (0.0–0.0)	0.0 (0.0–0.0)
90	4.3 (2.6–6.2)	0.0 (0.0–0.0)	95.7 (93.8–97.4)	0.0 (0.0–0.0)	0.0 (0.0–0.0)	0.0 (0.0–0.0)	0.0 (0.0–0.0)	0.0 (0.0–0.0)
183	2.1 (1.1–3.4)	1.9 (0.9–3.2)	36.5 (32.1–40.7)	35.2 (30.7–40.0)	24.0 (21.8–29.8)	0.0 (0.0–0.0)	0.0 (0.0–0.0)	0.2 (0.0–0.7)
365	0.6 (0.0–1.5)	3.0 (1.5–4.7)	28.4 (24.4–32.7)	13.4 (10.4–16.6)	45.3 (40.7–50.2)	5.2 (3.4–7.4)	3.4 (1.8–5.2)	0.7 (0.0–1.5)
548	0.0 (0.0–0.0)	3.6 (1.9–5.3)	14.1 (10.9–17.5)	7.7 (5.2–10.0)	56.5 (51.7–61.5)	11.1 (8.4–14.3)	6.3 (4.1–8.7)	0.9 (0.2–1.8)
730	0.0 (0.0–0.0)	3.6 (1.9–5.3)	6.7 (4.4–9.2)	3.9 (2.0–5.8)	66.4 (61.8–71.1)	11.4 (8.4–14.5)	7.1 (4.8–9.6)	0.9 (0.2–1.8)

**Table 2.** Proportion of Individuals With Composite Care Outcomes Across First 2 Years Following Linkage to PrEP Care

Time (d)	Disengaged From PrEP Clinic (2 + 5)	Prescribed PrEP and Continuously Engaged in Care (3 + 4)	Reinitiated on PrEP (6 + 7)	In Care and Current on Routine Labs (3 + 6)	In Care and Late for Routine Labs (4 + 7)	Engaged or Re-engaged in Care at PrEP Clinic (3 + 4 + 6 + 7)
0	0.0 (0.0–0.0)	79.4 (75.5–83.2)	0.0 (0.0–0.0)	79.4 (75.5–83.2)	0.0 (0.0–0.0)	79.4 (75.5–83.2)
7	0.0 (0.0–0.0)	86.8 (83.6–90.0)	0.0 (0.0–0.0)	86.8 (83.6–90.0)	0.0 (0.0–0.0)	86.8 (83.6–90.0)
30	0.0 (0.0–0.0)	93.0 (90.4–95.1)	0.0 (0.0–0.0)	93.0 (90.4–95.1)	0.0 (0.0–0.0)	93.0 (90.4–95.1)
90	0.0 (0.0–0.0)	95.7 (93.8–97.4)	0.0 (0.0–0.0)	95.7 (93.8–97.4)	0.0 (0.0–0.0)	95.7 (93.8–97.4)
183	25.9 (21.8–29.8)	71.7 (67.9–76.0)	0.0 (0.0–0.0)	36.5 (32.1–40.6)	35.2 (30.7–39.7)	71.7 (67.9–76.0)
365	48.3 (43.7–53.1)	41.8 (37.3–46.3)	8.6 (6.2–11.3)	33.6 (29.2–38.1)	16.8 (13.4–20.4)	50.4 (45.8–55.1)
548	60.1 (55.0–64.7)	21.7 (17.8–25.8)	17.3 (14.0–21.2)	25.1 (21.1–29.6)	13.9 (11.0–17.2)	39.0 (34.4–44.0)
730	70.0 (65.7–74.6)	10.6 (7.6–13.4)	18.5 (15.0–22.5)	18.1 (14.3–21.8)	11.0 (8.4–14.2)	29.0 (24.4–33.3)

**Figure 3.**
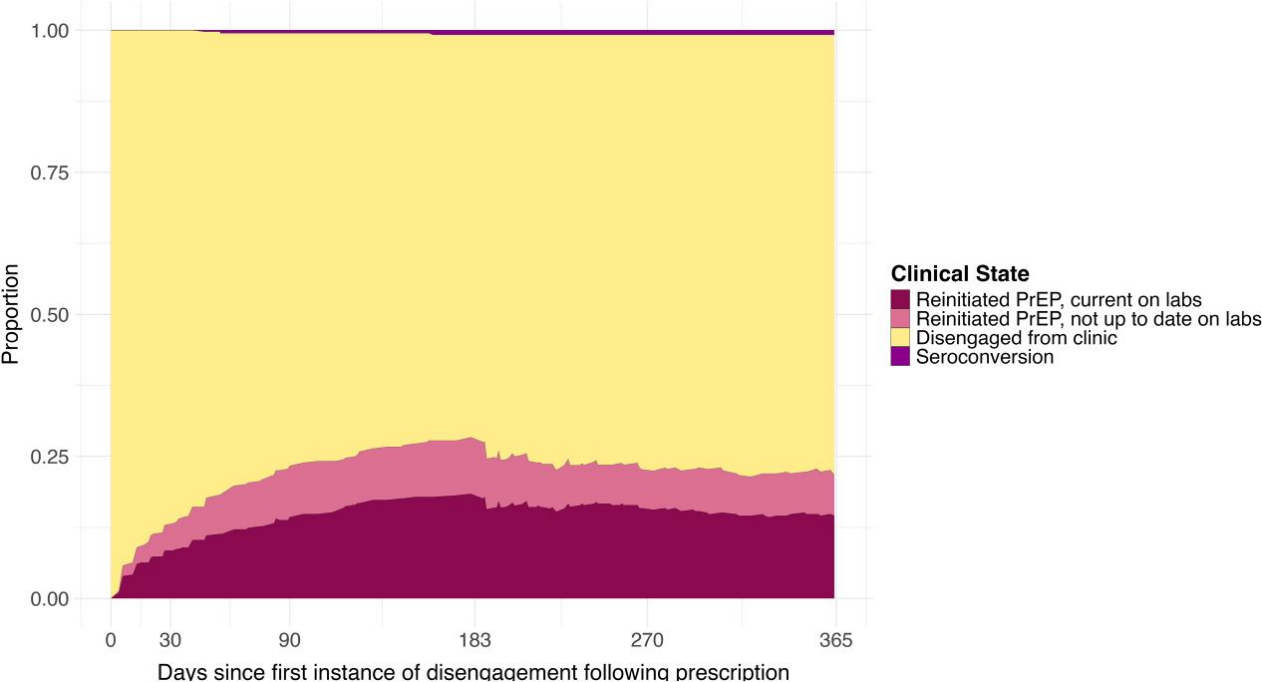
Clinical care outcomes in the first year following disengagement from care in a cohort of 470 people linked to PrEP care in St. Louis, Missouri between June 2014 and November 2021.

Of the 380 individuals who disengaged from the PrEP clinic at least once across the follow-up period, 12.9% (95% CI: 9.5–16.4) were re-engaged 30 days following disengagement; 28.3% (95% CI: 23.9–32.7) were re-engaged 6 months following disengagement (Figure 3; Supplementary Table 2).

Over the first year following linkage to PrEP care, users spent a restricted mean time of 238.0 days (95% CI: 230.1–246.4) continuously engaged in care and current on labs and 39.7 days (95% CI: 34.3–45.3) continuously engaged in care but late for lab screening (Supplmentary Table 3). In total, individuals spent a restricted mean time of 284.0 days (95% CI: 275.4–292.6) out of 365 days total engaged or re-engaged in care, and of those days, 240.9 (95% CI: 233.0–249.3) were current on lab testing (Supplementary Tables 3 and 4).

### Stratified Analyses of Disengagement From Care

**Figure 4.**
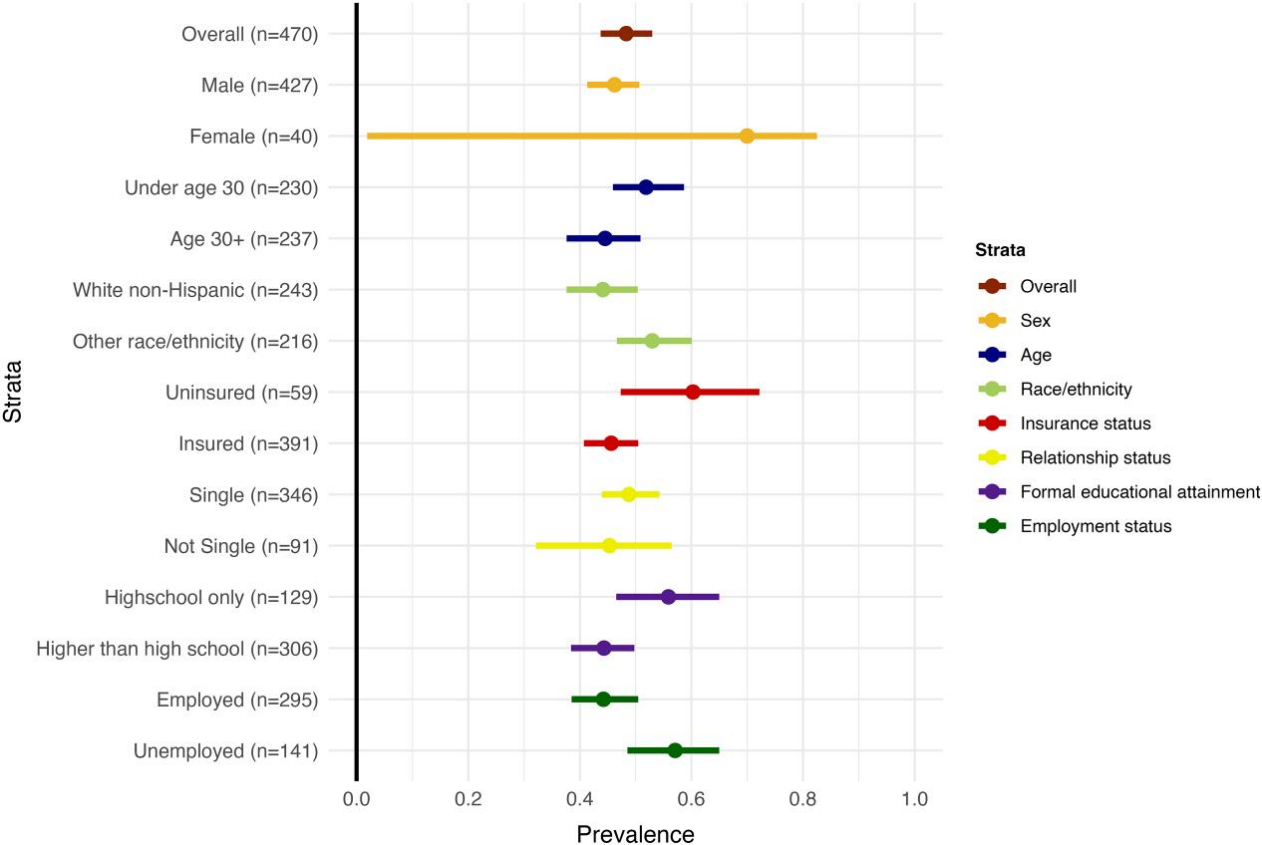
Stratified estimates of disengagement from PrEP care 1 year following linkage to care in a cohort of 470 people linked to Prep care in St. Louis Missouri between June 2014 and November 2021.

One year following linkage to PrEP care, females were more likely to be disengaged compared to males (70.0% [95% CI: 1.8–82.6] vs 46.2% [95% CI: 41.3–50.9]) as were those under the age of 30 compared to those 30+ (51.9% [95% CI: 45.6–58.3] vs 44.5% [95% CI: 37.7–50.9]); non-White or Hispanic individuals compared to White, non-Hispanic individuals (53.0% [95% CI: 45.8–59.4] vs 44.1% [95% CI: 38.2–50.3]); uninsured individuals compared to insured individuals (60.3% [95% CI: 47.2–72.9] vs 45.6% [95% CI: 40.6–50.3]); individuals with a high school education only compared to those with a higher than high school education (55.9% [95% CI: 46.9–64.6] vs 44.3% [95% CI: 38.7–49.7]); and unemployed individuals compared to employed individuals (57.1% [95% CI: 48.2–64.8] vs 44.2% [95% CI: 38.6–50.3]; Figure 4). Disengagement from the PrEP clinic 1 year following linkage to care was similar among those who reported being single and those who reported being in a relationship at enrollment (48.8% [95% CI: 43.3–54.2] and 45.3% [95% CI: 31.4–56.1], respectively; Figure 4).

## DISCUSSION

We characterized longitudinal engagement in clinical care and laboratory testing among individuals initiating PrEP care at two clinics in St. Louis, Missouri, an Ending the HIV Epidemic priority state, between June 2014 and November 2021 using multistate methods. While most individuals were prescribed PrEP the same day they were linked to care, approximately half had disengaged from care within the first year following linkage. Moreover, among those who remained in PrEP care, lab lapses were common with ∼35% of users late for at least one screening test 6 months following linkage to PrEP care and between 10–20% late for at least one screening test between 1 and 2 years following linkage. Disengagement from care differed across sociodemographic subgroups, including by sex assigned at birth, age, race/ethnicity, insurance status, formal educational attainment, and employment status, highlighting an urgent need for targeted PrEP persistence interventions.

Existing evidence suggests, globally, persistence on PrEP is poor in the first year following care initiation. A 2022 meta-analysis demonstrated that over 40% of individuals discontinued PrEP within the first 6 months [5, 24]. While retention in care in the first year following care initiation in this cohort was suboptimal, just 24% had disengaged by month 6 following enrollment, demonstrating comparatively better early-stage retention outcomes than what has been observed globally. Importantly, efforts were made to retain individuals using multiple modalities: clinic staff sent reminders via phone and the EHR messaging platform 1 week before an appointment; if a person wanted to reschedule their appointment they could do so via clinic schedulers, their provider, or PrEP navigators; if an individual missed their appointment, PrEP navigators contacted people three times to attempt to reschedule. Moreover, PrEP navigators routinely facilitated referrals for other health services and, during the COVID-19 pandemic, telehealth visits were rapidly implemented to offer alternative treatment seeking options. In line with existing evidence, these programmatic outcomes suggest appointment reminders, care navigation, and flexible visit types may support improved PrEP persistence in the first year following linkage to care [25, 26]. However, more personalized or resource-intensive interventions (eg, longitudinal one-on-one counseling; motivational interviewing) may be needed to support long term persistence [27, 28].

Disengagement from PrEP care differed across sociodemographic subgroups. Specifically, females, those under the age of 30, those from historically marginalized race and ethnicity groups (ie, those who were non-White or Hispanic), uninsured individuals, those with a high school education only, and those who were unemployed, were less likely to be engaged in care 1-year following linkage to PrEP care compared to their counterparts. These findings are supported by the results from a range of other studies which have shown that the hazard of PrEP discontinuation is significantly greater among females, younger persons, non-White individuals, and those with lower neighborhood-level SES compared to their respective counterparts [9, 29, 30]. This growing body of work suggests a need for targeted or tailored persistence-related supports for individuals from distinct subgroups. Additional research is needed to understand which interventions (eg, appointment reminders, peer navigation, etc.) work for whom and under what conditions.

Emerging PrEP technologies—including long-acting injectables (eg, long-acting injectable cabotegravir), extended-release oral formulations (eg, weekly and monthly oral lenacapavir), and subcutaneous implants—are promising alternatives to oral daily PrEP that may address a number of persistent barriers to PrEP uptake, adherence, and persistence in those who experience difficulty taking daily medications [31, 32]. However, numerous studies suggest individuals at heightened susceptibility to HIV continue to prefer daily oral PrEP over emerging prevention technologies [33, 34]. It is also critical to consider the potential public health impact of less frequent clinic visits among those using long-acting versus once-daily oral prevention technologies, including missed opportunities for monitoring and treating STIs and chronic care conditions (eg, hypertension, diabetes). Notably, extended intervals between STI testing among PrEP users has been shown to delay STI diagnosis and treatment, contributing to increasing STI transmission rates [35]. The complexity of injection administration and follow-up appointments, potential for breakthrough infections or resistance-associated mutations, and implementation of Trump-era policies that target the elimination of HIV prevention and care programs and funding, may further hinder the viability of rapid scale-up of these emerging tools [31, 36, 37]. Broadly, direct, effective person-centered conversations regarding preferences and the relative benefits and drawbacks of each PrEP prevention technology will be paramount to improving individual experience and adherence outcomes, including PrEP persistence. Such practices will be particularly pertinent in priority settings such as Missouri where PrEP use continues to lag national estimates [38].

It is important to note that this study was conducted in two academic infectious disease clinics in the greater St. Louis area, where a majority of individuals initiating PrEP were White, insured, and college-educated males. The limited geographic scope of our recruitment may impede the generalizability of our study findings to community-based, rural, and under-resourced settings where the sociodemographic profile of PrEP initiators may differ (eg, a higher proportion of uninsured initiators) [39]. Moreover, recent evidence suggests PrEP uptake by race, ethnicity, sex, and region changed significantly over time between 2012 and 2021 [40]. While data from other PrEP cohort studies conducted in the United States have had a similar sociodemographic makeup to our cohort [9], there is a clear need for more nuanced, recent engagement data to understand how outcomes may differ over time and among underrepresented PrEP initiators.

This study has limitations. It is well documented that individuals discontinue PrEP for myriad reasons [19, 41], including behavioral changes that alter PrEP users' susceptibility to HIV and, thus their clinical indication. We were unable to ascertain reasons for PrEP discontinuation and could not adequately differentiate instances of disengagement that were versus were not clinically indicated. Moreover, because we lost contact with users after disengagement, we could not determine whether they reinitiated PrEP care with a new provider. Literature indicates silent transfers are common in the care-seeking journey of people with HIV [42, 43]; such behaviors may be similarly frequent among those seeking HIV prevention services, though an absence of data precludes our understanding of such commonalities. While there has been some effort to contact PrEP users who disengage from care to ascertain true outcomes, success has been limited [19, 44]. One study reported finding just 15% of individuals who disengaged from PrEP care at their clinic, with 32% (ie, <5% of the total population) of those who were found reporting receipt of PrEP services from a new provider [44]. Future research should employ double-sampling methods that are more common in engagement in HIV care research to verify outcomes, including silent transfers, among PrEP users [45–47]. In addition to these limitations, 179 individuals had follow-up time (including 58 who were enrolled after 15 March 2020) during the COVID-19 pandemic, which has been shown to impact provision and uptake of HIV services in the United States and globally. Numerous measures were put in place to promote flexibility in care-seeking during the height of the pandemic, including telehealth visits and mail-in STI monitoring. As such, it is possible that treatment outcomes among those initiating or seeking PrEP care during COVID-19 differed from those enrolled and followed prior to this period. While a sensitivity analysis exploring differential outcomes among those initiated PrEP care before and after 15 March 2020 is warranted, the comparatively small number of individuals initiating care after this time point limited power to undertake such analyses. Additional research is needed to explore how changes in clinic practices, such as the provision of telehealth visit options, impact longitudinal engagement in PrEP care. Finally, prescription fill data were unavailable, limiting our ability to characterize medication use. Estimates of individuals “prescribed PrEP” should be interpreted with caution as this outcome may not reliably capture individuals taking PrEP.

## CONCLUSIONS

Lapses in clinic visits and routine HIV and STI screening across the first two years following care initiation were common among those seeking PrEP care in the Greater St. Louis area between 2014 and 2021. Moreover, a majority of people who disengaged from care did not return. Females, uninsured individuals, and other historically marginalized groups were particularly susceptible to poor persistence outcomes, suggesting tailored PrEP persistence interventions are warranted. Multistate methods can capture dynamic PrEP engagement patterns and should be used in planning and evaluation activities to help tailor public health programming related to PrEP care.

## Supplementary Material

ofaf795_Supplementary_Data

## Supplementary Data

Supplementary materials are available at *Open Forum Infectious Diseases* online. Consisting of data provided by the authors to benefit the reader, the posted materials are not copyedited and are the sole responsibility of the authors, so questions or comments should be addressed to the corresponding author.
