# Supplementary material for: PrEParing for Long-acting Technologies: A Multistate Analysis of PrEP Persistence and HIV and STI Lab Coverage Among Oral PrEP Initiators in St. Louis, Missouri (2014–2021)
Source: Open Forum Infect Dis. 2026 Jan 9;13(1):ofaf795. doi: 10.1093/ofid/ofaf795 (PMC12809563; doi:10.1093/ofid/ofaf795)
Supplement: ofaf795_Supplementary_Data [file ofaf795_supplementary_data.docx]

**Supplemental Digital Content**

[Supplemental Table 1. Sociodemographic characteristics of 470 individuals initiating pre-exposure prophylaxis care at the Washington University Infectious Disease Clinic between June 2014 and November 2021 2](#_Toc218853051)

[Supplemental Table 2. Proportion of individuals in distinct care states across the first year following disengagement from the PrEP clinic 3](#_Toc218853052)

[Supplemental Table 3. Restricted mean time spent in each mutually exclusive and exhaustive PrEP care continuum state at Year 1 and Year 2 following linkage to PrEP Care 3](#_Toc218853053)

[Supplemental Table 4. Restricted mean time spent in composite care states at Year 1 and Year 2 following linkage to PrEP Care 3](#_Toc218853054)

Supplemental Table 1. Sociodemographic characteristics of 470 individuals initiating pre-exposure prophylaxis care at the Washington University Infectious Disease Clinic between June 2014 and November 2021

|  | **N (%)** |
| --- | --- |
| **Sex assigned at birth** |  |
| Male | 427 (91.4) |
| Female | 40 (8.6) |
| Missing | 3 |
| **Age (Median/interquartile range)** | 29 (18, 73) |
| Under 30 | 230 (49.3) |
| 30+ | 237 (50.7) |
| Missing | 3 |
| **Race/Ethnicity** |  |
| White, non-Hispanic | 243 (52.0) |
| Black, non-Hispanic | 136 (29.1) |
| Hispanic | 31 (6.6) |
| Asian | 27 (5.8) |
| Other race/ethnicity | 22 (4.7) |
| Missing | 3 |
| **Relationship status** |  |
| Married/partnered | 38 (8.7) |
| Single, never married | 346 (74.4) |
| Other | 53 (16.9) |
| Missing | 33 |
| **Highest Education** |  |
| ≤High School | 129 (29.7) |
| College+ | 306 (70.3) |
| Missing | 35 |
| **Employment** |  |
| Unemployed | 35 (8.0) |
| Employed | 295 (67.7) |
| Student | 77 (17.7) |
| Other | 29 (6.7) |
| Missing | 34 |
| **Insurance** |  |
| Uninsured | 59 (13.1) |
| Insured (Public or Private) | 391 (86.9) |
| Missing | 20 |

Supplemental Table 2. Proportion of individuals in distinct care states across the first year following disengagement from the PrEP clinic

| Time  (days) | Disengaged from PrEP clinic | Re-initiated on PrEP and current on routine labs | Re-initiated on PrEP and late on routine | HIV infection | Composite: Re-initiated on PrEP |
| --- | --- | --- | --- | --- | --- |
| 30 | 87.1 (83.6, 90.5) | 8.4 (5.8, 11.3) | 4.5 (2.4, 6.6) | 0.0 (0.0, 0.0) | 12.9 (9.5, 16.4) |
| 90 | 76.1 (71.9, 80.6) | 14.3 (11.1, 17.8) | 9.0 (6.1, 11.7) | 0.5 (0.0, 1.0) | 23.3 (19.0, 27.5) |
| 183 | 70.8 (66.3, 75.2) | 18.4 (14.9, 22.2) | 9.9 (6.8, 12.8) | 0.8 (0.0, 1.9) | 28.3 (23.9, 32.7) |
| 270 | 76.5 (72.3, 80.6) | 15.9 (12.4, 19.6) | 6.8 (4.3, 9.7) | 0.8 (0.0, 1.9) | 22.7 (18.7, 26.8) |
| 365 | 77.4 (73.1, 81.6) | 14.6 (11.1, 18.1) | 7.2 (4.6, 10.0) | 0.8 (0.0, 1.9) | 21.7 (17.5, 26.1) |

Supplemental Table 3. Restricted mean time spent in each mutually exclusive and exhaustive PrEP care continuum state at Year 1 and Year 2 following linkage to PrEP Care

| Year | Linked to PrEP Clinic (1) | Disengaged from care prior to PrEP prescription (2) | Prescribed PrEP and current on routine labs (3) | Prescribed PrEP and late for routine labs (4) | Disengaged from PrEP clinic following PrEP initiation (5) | Re-initiated on PrEP and current on routine labs (6) | Re-initiated on PrEP and late on routine labs (7) | Seroconverted (8) |
| --- | --- | --- | --- | --- | --- | --- | --- | --- |
| 1 | 12.4 (8.1, 17.0) | 4.7 (2.4, 7.2) | 238.0 (230.1, 246.4) | 39.7 (34.3, 45.3) | 63.3 (56.2, 70.3) | 2.9 (1.6, 4.5) | 3.4 (1.8, 5.2) | 1.0 (0.0, 2.3) |
| 2 | 13.0 (8.3, 18.1) | 17.4 (9.5, 25.8) | 290.5 (275.9, 306.0) | 70.6 (60.4, 71.6) | 271.6 (251.3, 293.4) | 38.4 (30.2, 47.6) | 24.9 (18.4, 32.3) | 4.0 (0.5, 8.2) |

Supplemental Table 4. Restricted mean time spent in composite care states at Year 1 and Year 2 following linkage to PrEP Care

| Year | Disengaged from PrEP clinic (2+5) | Prescribed PrEP and continuously engaged in care (3+4) | Re-initiated on PrEP (6+7) | In care and current on routine labs (3+6) | In care and late for routine labs (4+7) | Engaged or re-engaged in care at PrEP clinic (3+4+6+7) |
| --- | --- | --- | --- | --- | --- | --- |
| 1 | 68.0 (60.8, 75.0) | 277.7 (269.3, 286.1) | 6.3 (4.1, 8.8) | 240.9 (233.0, 249.3) | 43.1 (38.0, 49.1) | 284.0 (275.4, 292.6) |
| 2 | 289.0 (268.3-308.6) | 361.1 (342.9-378.6) | 63.43 (52.9-76.0) | 328.9 (312.3-347.9) | 95.5 (83.5-108.0) | 424.4 (404.9-446.0) |
